# Clinicopathological and molecular characterizations of pulmonary NUT midline carcinoma

**DOI:** 10.1002/cam4.4096

**Published:** 2021-08-19

**Authors:** Mian Xie, Xinge Fu, Wei Wang

**Affiliations:** ^1^ Department of Medical Oncology Guangdong Provincial People's Hospital and Guangdong Academy of Medical Sciences Guangzhou China; ^2^ Department of Pathology The First Affiliated Hospital of Guangzhou Medical University Guangzhou China; ^3^ Department of Thoracic Surgery The First Affiliated Hospital of Guangzhou Medical University Beijing China

**Keywords:** lung cancer, NUT midline carcinoma, squamous cell carcinoma

## Abstract

**Introduction:**

Pulmonary nuclear protein of the testis (NUT) midline carcinoma (NMC) is a aggressive cancer with t (15, 19) translocation. Here we present the clinicopathological characteristics and molecular genetics alterations of primary pulmonary NMC.

**Methods:**

Fluorescence in situ hybridization (FISH) assay was performed to evaluate *NUT* translocation. Next generation sequencing (NGS) was performed to investigate genomic landscape. A panel of 289 lung cancer tissues with undifferentiation was retrospectively screened for *NUT* expression by immunohistochemical (IHC) assay.

**Results:**

Overall, 2136 lung cancer samples were reviewed. We consecutively identified 12 cases of primary pulmonary NMC. Computed tomography revealed centrally located bulky lung mass with ipsilateral mediastinal lymph node and pleural involvements. Tumor cells presented diffuse poor differentiation and focal squamous differentiation with positive *NUT* expression. *NUT* rearrangement was confirmed by FISH assay. Ten NMC samples were investigated by NGS. The most common alterations identified were *P53*, *PIK3CA*, *AUTS2*, *ITIH2*, and *CDKL5* genes. Pulmonary NMC exhibited increased activity of PI3K/AKT pathway. In the screening study, *BRD4*‐*NUT* rearrangement was identified in two cases.

**Conclusion:**

*NUT* rearrangement remains the gold standard in the diagnosis of pulmonary NMC. PI3K inhibition is a potential targeted therapy for pulmonary NMC.

## Graphical abstract

We present the clinicopathological characteristics and molecular genetics alterations of primary pulmonary NUT midline carcinoma (NMC). Ten NMC samples had retrospectively undergone comprehensive genomic profiling (CGP), which revealed increased activity of the PI3K/AKT pathway. PI3K inhibition may be beneficial for BRD‐NUT fusion‐positive pulmonary NMC.

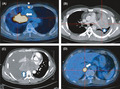

## 1 INTRODUCTION

Nuclear protein of the testis (NUT) midline carcinoma (NMC) is a rare, aggressive undifferentiated tumor.^1^, ^2^, ^3^ NMC is characterized with a rearrangement of *NUT* (Nut midline carcinoma) gene on chromosome 15, leading to the fusion of bromodomain containing (*BRD*) or specific variants.^4^ An immunohistochemical assay using NUT antibody contributes to the diagnosis of NMC.^5^ Most of NMC patients progressed rapidly to death. The pathogenesis of NMC remains unknown. NMC represent an aggressive tumor in the midline organs.^6^, ^7^ A few cases of pulmonary NMC were reported.^8^, ^9^ The molecular findings of pulmonary NMC is poorly defined.

The vast majority of patients with NMC were treated with multimodal therapy including surgery, radiation therapy, and chemotherapy. Unfortunately, the outcome of most patients with NMC remains poor, with a median overall survival of only 6.7 months.^10^ Some chemotherapeutic agents have been tried—including the use of doxorubicin‐based regimens—unfortunately, they are not associated with improved outcomes. In the largest reported retrospective analysis of 63 patients with NMC, early radiation therapy and extent of surgery were the only predictors of improved survival. No chemotherapy regimen was shown to improve survival.^4^ Histone deacetylase (HDAC) or bromodomain and extra terminal (BET) inhibitor induces squamous differentiation of NMC cells.^3^, ^11^, ^12^, ^13^ Here we present the clinicopathological characteristics and genetic alterations of pulmonary NMC. We also retrospectively screened undifferentiated non‐small cell lung cancer (NSCLC) cases for NUT expression by immunohistochemistry assay (IHC).

## 2 PATIENTS AND METHODS

### 2.1 Patients

Pulmonary NMC were consecutively diagnosed in Guangzhou Medial University and Sun yat‐sen University between 2014 and 2017 after surgical resection or bronchoscopy examination. Follow‐up data were obtained from electronic medical records. This study was reviewed by institutional review board (IRB). Informed consent was obtained from the patients.

### 2.2 Radiological examination

The patients underwent contrast‐enhanced chest CT to evaluate distant metastasis in the thorax and regional lymph nodes. Before the administration of 18F‐FDG PET/CT, at least 6 h of fasting time was required and blood glucose levels of all the patients were checked. Before PET scan, a low‐dose CT scan without contrast enhancement was conducted from the skull vertex to the knee level under supine position with quiet respiration for attenuation correction. Consequently, PET/CT scans were performed using a 64‐slice CT Discovery^®^ PET/CT 600 or 690 apparatus (General Electric Healthcare).

### 2.3 Immunohistochemical assay (IHC)

IHC was performed on formalin‐fixed, paraffin‐embedded (FFPE) sections using the Envision Plus detection system (Dako). CK5/6 (Santa Cruz), thyroid transcription factor‐1 (TTF‐1) (Santa Cruz), P63 (Santa Cruz) and NUT (C5261, Cell Signaling Technology) were used as primary antibodies. IHC staining was reviewed by two pathologists. Strong staining of nucleus in more than 50% tumor cells was regarded as NUT positive.^5^ Fluorescence In Situ Hybridization (FISH)

Dual‐color FISH assays were conducted to evaluate *NUT* translocation on tumor sections. Probes of NUT were BAC clones 1H8 and 64o3 and centromeric clones 412e10, and 3d4. Over 80% hybridized efficiency in the section was regarded as interpretable.

### 2.4 Screening study

NUT expression was investigated by IHC assay in a panel of 289 NSCLC undifferentiated tumor tissues obtained between 2011 and 2012. Tumors diagnosed as lung adenocarcinoma and small‐cell lung cancer (SCLC) were excluded. FISH assay was performed to detect NUT translocation in NUT overexpression tumor.

### 2.5 Next generation sequencing (NGS)

Next generation sequencing was performed in an Illumina Hiseq 2500 platform to confirm the mutated genes.^14^ Hybrid capture of 16 introns and 439 exons from 341 cancer‐related genes was conducted. The hybridized capture library was sequenced to >500 × average unique coverage. Somatic gene mutations and copy number alterations were identified.

### 2.6 PI3K/AKT signaling pathway PCR assay

The activational level of PI3K/AKT signaling was checked by PI3K/AKT signaling PCR Array kits (Qiagen).

## 3 RESULTS

### 3.1 Clinical characteristics

Overall, 2136 lung cancer samples were reviewed. Twelve primary pulmonary NMC patients were consecutively diagnosed. The clinical characteristics are shown in Table 1. The median age of the patients at diagnosis was 39 years old (range 22−74). Of these, four patients were nonsmokers. All patients complained of over 1 month of cough, hemoptysis, and shortness of breath. One patient with locally advanced disease received pneumonectomy, mediastinal lymphadenectomy, and carina resection following by intraoperative radiotherapy of diaphragm. Other eleven patients received chemotherapy or radiotherapy followed by chemotherapy. Chemotherapeutic agents included platinum plus albumin‐bound paclitaxel (abraxane) or doxorubicin. In two patients, chemotherapy achieved transient stable disease and no subsequent treatments achieved clinical benefit. Partial response was observed in Case 5 after two cycles of chemotherapy with abraxane and cisplatin (day 40). After three cycles of chemotherapy, the disease progressed (day 70). The patient refused radiotherapy and he received palliative care (day 100) (Figure S1). The median survival time was 5.9 months (Table 1).

**TABLE 1:** Clinical characteristics of pulmonary NUT midline carcinoma

	No of patients (%)
Median age (range)	39 (22−74)
Gender	
Male	10 (83.3%)
Female	2 (16.7%)
Smoking history	
Smoker	8 (66.7%)
Nonsmoker	4 (33.3%)
Primary site	
LUL	3 (25.0%)
RUL	3 (25.0%)
LLL	4 (33.3%)
RLL	2 (16.7%)
Tumor size (range)	7.2 (4.0−12.7)
Distant metastasis	
Yes	8 (66.7%)
No	4 (33.3%)
Treatment	
Surgery	1 (8.3%)
CR	5 (41.7%)
C	6 (50.0%)
Median survival time (mon)	5.9 (2.3−11.5)

Abbreviations: C, chemotherapy; CR, chemotherapy and radiotherapy; LLL, left low lobe; LUL, left upper lobe; RLL, right low lobe; RUL, right upper lobe.

### 3.2 Radiological examination

**FIGURE 1:**
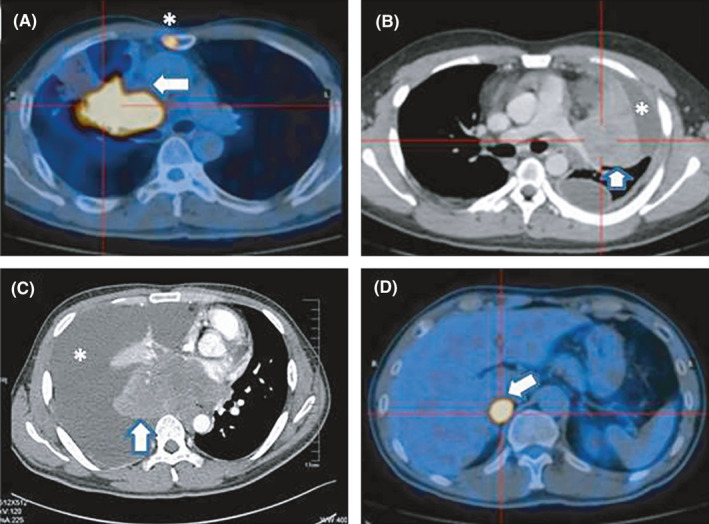
CT and PET‐CT images of pulmonary NUT midline carcinoma (NMC). (A) Pulmonary tumor in the right upper lobe (arrow) with SUV 18.6. Sternum metastasis is shown (asterisk). (B) Pulmonary tumor in the left upper lobe (arrow) with malignant pleural effusion (asterisk). (C) Pulmonary tumor in the right low lobe (arrow) with malignant pleural effusion (asterisk). (D) Adrenal metastasis with SUV 17.8 (arrow)

All patients received computed tomography (CT) and/or positron emission tomography (PET)/CT when diagnosed. The primary lung tumors were centrally located and shown as large masses (5−12.7 cm) (Figure 1A–C). Half of the patients had malignant pleural effusion (Figure 1B–C). Bilateral mediastinal lymphadenopathy and supraclavicular nodal metastasis were identified in seven patients. SUV (standard uptake value) of 18F‐FDG in primary lung mass was over 10 among seven patients in PET/CT examination. Distant metastasis included bone (Figure 1A), adrenal (Figure 1D), subcutaneous soft tissue of backside, liver, and brain.

### 3.3 Pathological findings

**FIGURE 2:**
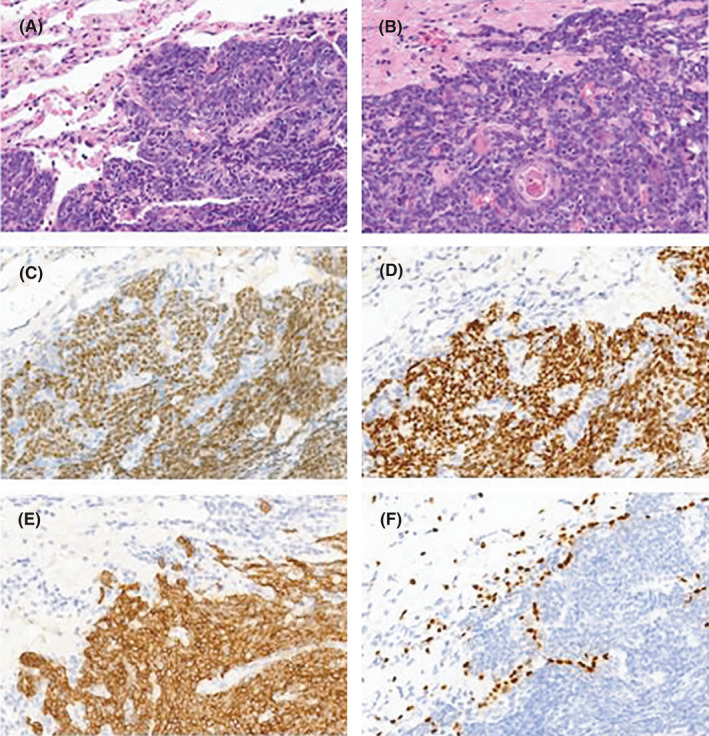
Morphology and immunohistochemical stain of pulmonary NMC. (A–B) Hematoxylin–eosin‐stained slide at 400 ×. Positive expressions of NUT (C), P63 (D), CK5/6 (E) and negative expression of TTF‐1 (F) in tumor cells

The tumors consisted of undifferentiated small cells with focal squamous differentiation. Squamous differentiation represented keratinized cytoplasm dispersed in the tumor cells (Figure 2A–B). Immunohistochemistry showed that positive nuclear staining of *NUT* (Figure 2C) and *P63* (Figure 2D) protein in tumor cells. All cases in the series showed cytoplasmic expression of *CK5*/*6* protein in tumor cells (Figure 2E, all panels at 400 **×**). *TTF*‐*1* expression was negative in tumor cells and positive nuclear staining in normal alveolar epithelial cells in eleven of twelve cases (Figure 2F). Histopathological features are summarized in Table 2.

**TABLE 2:** Pathological findings in patients with pulmonary NUT midline carcinoma

	NUT		Immunohistochemistry		
Patient	IHC	FISH	P63	CK5/6	TTF−1
1	+	+	+	+	−
2	+	+	+	+	−
3	+	+	+	+	−
4	+	−	+	+	−
5	+	+	+	+	+
6	+	+	+	+	−
7	+	+	+	+	−
8	+	+	+	+	−
9	+	+	+	+	−
10	+	+	+	+	−
11	+	+	+	+	−
12	+	+	+	+	−

+, positive expression; −, negative expression.

### 3.4 Molecular characterizations of pulmonary NMC

**FIGURE 3:**
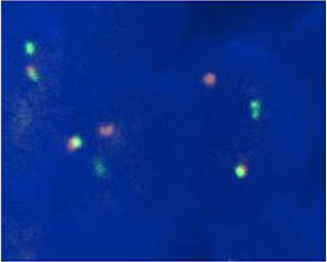
*BRD4*‐*NUT* translocation identified by FISH assay. Shown is dual color bring together FISH on tumor cell nuclei using red and green probes covering *BRD4* and *NUT* genes, respectively. Nuclei with red‐green doublets demonstrate fusion of *BRD4* and *NUT*

**FIGURE 4:**
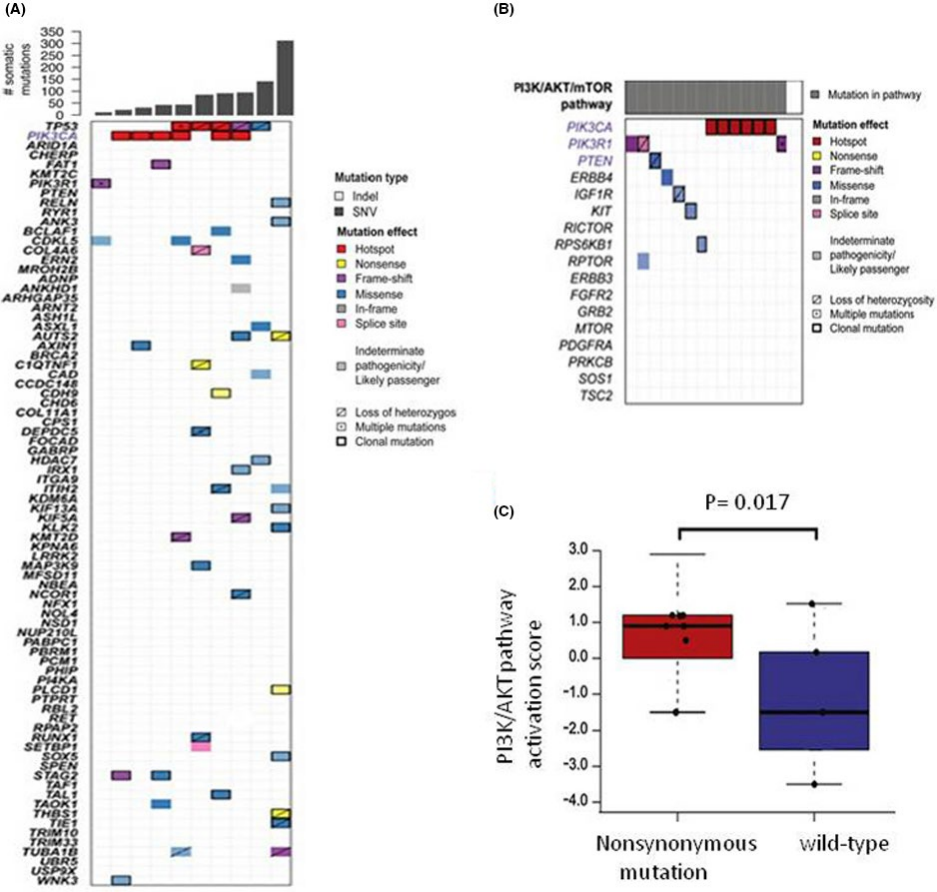
Next generation sequencing (NGS) in pulmonary NMC. (A) Somatic nonsynonymous mutations of pulmonary NMC. (B) Mutated genes related to PI3K/AKT pathway in pulmonary NMC. (C) PI3K/AKT pathway activity scores between pulmonary NMC with somatic mutations and wild‐type tumors

In order to identify the fusion partner of the putative NUT rearrangement, we subsequently performed *NUT* fusion in situ hybridization. *BRD4*‐*NUT* rearrangement was confirmed in 11 patients (91.7%) (Figure 3; Table 2). Next generation sequencing was performed in 10 pulmonary NMC tumors. Common nonsynonymous mutated genes were *P53*, *PIK3CA*, *AUTS2*, *ITIH2*, and *CDKL5* (Figure 4A). Somatic mutations of *P53* included *P53 G245N*, *P53 H179Y*, P53 R196*, P53 Q136*, P53 R213*, P53 Q100*, and *P53 G266R*. *PIK3CA* gene was activated missense mutation and cancer hotspots were E542K, and H1047R. Other recurrently mutated genes consisted of *BCLAF1*, *FAT1*, *TUBA1B*, and *ANK3*.

In order to identify the affected signaling pathway in pulmonary NMC, mutated genes found in NGS assay were mapped to KEGG pathways. The result showed that 60% (6/10) of pulmonary NMC had somatic nonsynonymous mutation in PI3K/AKT pathway, including *PTEN*, *PIK3R1*, *PIK3CA*, *ERBB4*, and *RPTOR* (Figure 4B).

Moreover, patients with somatic mutation in PI3K/AKT pathway had significantly worse overall survival than patients without gene mutations in this pathway (*p* = 0.013) (Figure S2). Since NGS assay showed somatic mutated genes were associated with PI3K/AKT pathway, PI3K/AKT pathway PCR array was conducted to identify whether nonsynonymous mutations contributed to the activation of PI3K/AKT pathway. The results indicated that pulmonary NMC with somatic mutations had higher activation scores of PI3K/AKT pathway than wild‐type tumors (*p* = 0.017) (Figure 4C).

### 3.5 Screening study of pulmonary NMC in non‐small lung cancer (NSCLC) cohort

NUT positive staining was identified in two cases by IHC assay among 289 patients. *BRD4*‐*NUT* rearrangement was confirmed by FISH assay. The patients were both nonsmoking young men with history of cough and hemoptysis. CT scan showed bulky mass in the upper lobe with liver and bone metastases. The patients were diagnosed as stage IIIB NSCLC and received chemotherapy with pemetrexed and cisplatin. However, they died rapidly 3 months after diagnosis.

## 4 DISCUSSION

We present extensive clinicopathological and molecular characteristics of pulmonary NMC. To our knowledge, this is the largest scale of retrospective study on pulmonary NMC. This result provides the detailed clinical related findings which might serve as a useful reference for the treatment of this rare thoracic cancer.

In our study, the median age of pulmonary NMC patients was 39 years old, younger than the other reports.^1^, ^8^, ^9^ The overall survival time of young patients (range 2.0−2.2 months) was shorter than that among older patients (range 4.0−6.9 months), suggesting the poor prognosis in younger pulmonary NMC patients. Distinct from the previous studies, the smoking history (light and heavy) commonly existed in our study.^8^, ^9^ Thus, young patients with smoking history should not be excluded from the differential diagnosis of pulmonary NMC. The expression of EBER was negative in our study, which reflected that Epstein–Barr virus does not play an important role in the pathogenesis of pulmonary NMC. Pulmonary NMC occurs in less than 1% of lung cancer. Lung cancer with morphology of adenocarcinoma should be excluded from differential diagnosis of NMC since NMC is considered among non‐gland forming epithelioid tumors.

NMC typically arises from midline anatomic sites. All cases presented with centrally located large lung mass, often with ipsilateral pleural involvement. Notably, although brain metastasis was not shown at the time of diagnosis, one patient developed brain metastasis during the disease progressed. The ipsilateral lung metastasis was also observed in one patient. Together with these radiographic findings, there was no specific route of metastasis in pulmonary NMC.

The outcome of primary NMC is poor and no standard treatment regimen is available so for. Chemotherapy or chemoradiotherapy was performed in the most reported cases with advanced stage disease and no chemotherapeutic agent showed beneficial.^1^, ^15^, ^16^, ^17^ In this study, although one patient achieved partial response after two cycles of therapy with albumin‐bound paclitaxel plus cisplatin, he rapidly developed progressive disease after the third cycle of chemotherapy. Due to the aggressive growth of the tumor, completely surgical resection is not feasible. In approximately 75% of the NMC patients, NUT was fused with BRD4.^4^, ^18^, ^19^ In this case serials, 91.7% of pulmonary NMC had *BRD4*‐*NUT* fusion. *BRD4*‐*NUT* fusion oncoprotein activates *p300*, leading to hypoacetylation and inadequate expression of genes for differentiation.^20^, ^21^ Histone deacetylase inhibitor (HDACi) restores chromatin acetylation and induces differentiation of NMC cells. BET inhibitor (BETi) GSK 525762 competitively inhibits bromodomain‐acetyl‐histone binding and induces squamous differentiation of NMC cells. Phase I study of GSK 525762 treating NMC is still recruiting. Our study revealed that pulmonary NMC patients with somatic mutation in PI3K/AKT pathway had significantly worse overall survival, providing a genetic basis for the efficacy of PI3K/AKT pathway inhibitor. The results support the recent preclinical observation that dual HDAC and PI3K inhibition may be beneficial for *BRD*‐*NUT* fusion‐positive NUT midline carcinoma (NMC).^22^

The pathologic findings of pulmonary NMC are consistent with a poorly differentiated carcinoma with focal squamous differentiation. We noted the expressions of CK5/6 and p63 were evident, while expressions of TTF‐1 expression in the tumor cells were negative in the most cases. TTF‐1 is expressed in alveolar type II cells and in a few bronchial and bronchiolar basal cells in normal lung tissues. Diffuse positivity for both P63 and TTF‐1 in Case 5 suggested that the tumor cells were derived from basal cells or have the characteristics similar to basal cells of the bronchiolar epithelium.^8^ Extensive P63 expression with focal TTF‐1 in the same tumor cell population is not common in lung cancer and should take the diagnosis as NMC into consideration.

Pulmonary NMC progressed rapidly after treatment of surgery, chemotherapy, or chemoradiotherapy. Computed tomography showed centrally located large lung mass with ipsilateral mediastinal lymph node and pleura involvements. Tumor cells presented diffuse poorly differentiation and focal squamous differentiation with positive expression of P63 and CK5/6. Positive staining of NUC IHC contributes to the diagnosis of NMC, however, NUT rearrangement evaluated by FISH remains the gold standard of diagnosis in this unique population. Proper diagnosis and effective treatment algorithm should be made for pulmonary NMC in the future.

In this study, however, the small scale of retrospective study limits the findings of molecular characterizations of pulmonary NMC. Further investigations of pulmonary NMC are needed at both preclinical and clinical levels. Pulmonary NMC is uniformly and rapidly lethal and thus prompt recognition is necessary to permit proper patient counseling and hopefully to eventually offer effective therapy.

## ETHICS APPROVAL AND CONSENT TO PARTICIPATE

The study was approved by the institutional review boards of Guangdong Provincial People's Hospital and The First Affiliated Hospital of Guangzhou Medical University. All patients provided informed written consent to participate prior to sample collection.

## CONFLICT OF INTEREST

None declared.

## CONSENT TO PUBLICATION

Written consent was collected from each patient for future data publication.

## Supporting information

Fig S1

Fig S2

## Data Availability

The authors confirm that the data supporting the findings of this study are available within the article.
