# Supplementary figures and images for: Clinicopathological and molecular characterizations of pulmonary NUT midline carcinoma
Source: Cancer Med. 2021 Aug 19;10(17):5757–64. doi: 10.1002/cam4.4096 (PMC8419746; doi:10.1002/cam4.4096)

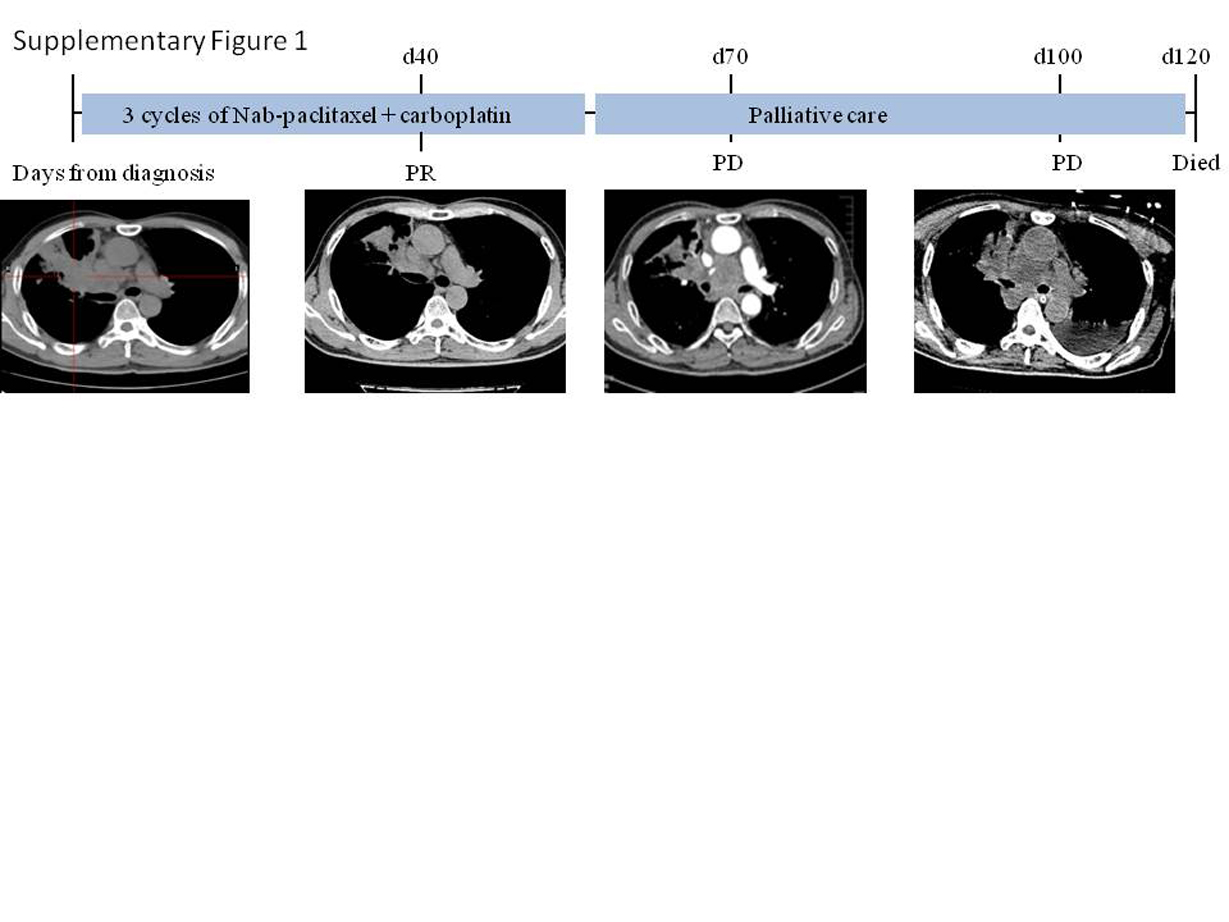

Supplement: Supplementary file 1 — Fig S1 [file CAM4-10-5757-s002.jpg]

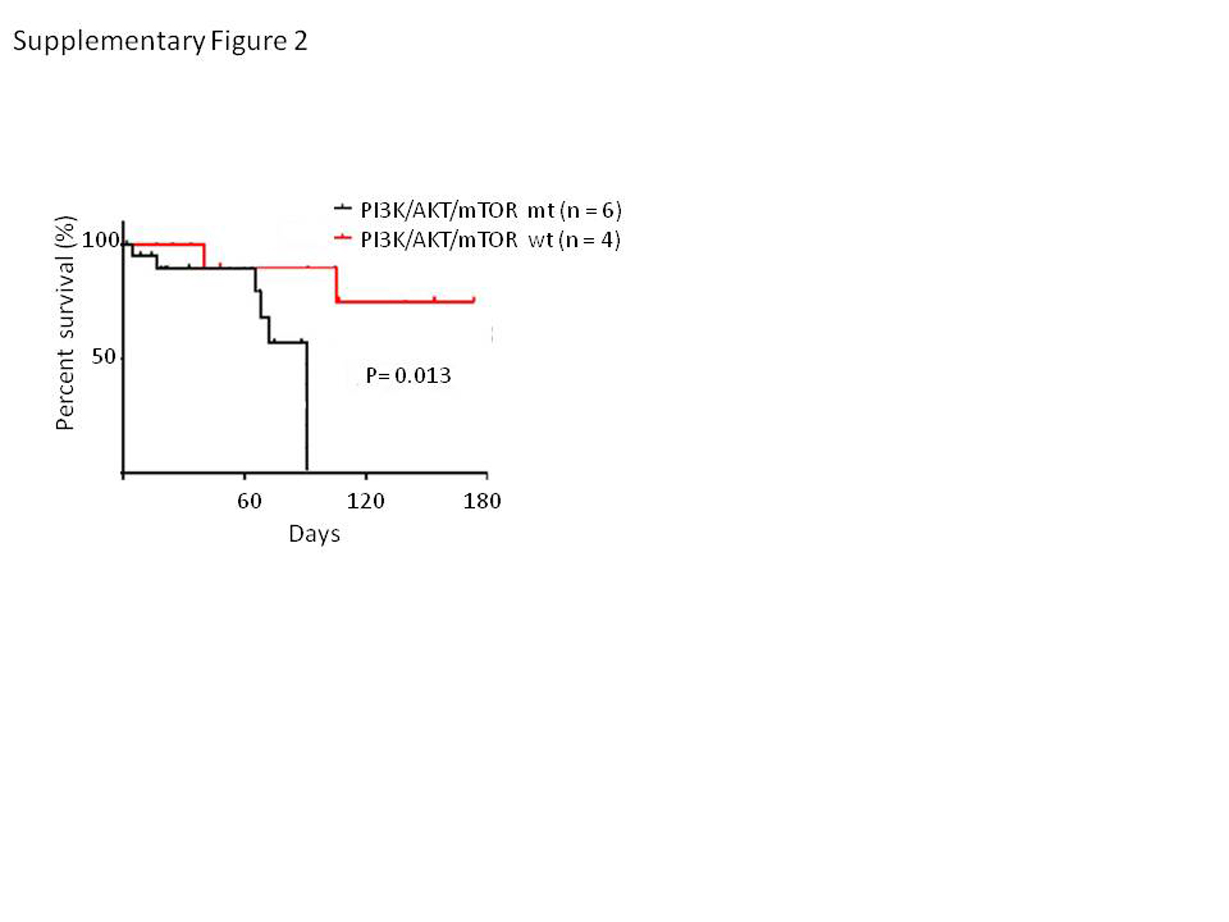

Supplement: Supplementary file 2 — Fig S2 [file CAM4-10-5757-s001.jpg]
